# Examination of the Body of Dr. Edson, Reported at a Meeting of the New York Pathological Society

**Published:** 1847-05

**Authors:** 


*Examination of the body of Dr. Edson, reported at a meeting of the New York Pathological Society. From the New York Annalist.*

Dr. Sabine exhibited the specimen taken from the body of the late Dr. Edson, and read the following account of the post mortem examination, made by him from his note book :

Feb. 14th.—Examined the body of Dr. Edson, who has been recently exhibiting in this city as an extraordinary emaciated subject —the brother of Calvin Edson, who was exhibited many years ago as the living skeleton.

The external appearance of this subject differed materially from the emaciation of tubercular disease. The body and limbs were very small, but round and firm ; the face slightly sallow. I should suppose the body would weigh 80 or 90 lbs. On cutting down to the superficial fascia over the abdomen, the fat was about the thickness of a dollar, covering the muscles ; the muscles firm and healthy ; stomach small, and situated entirely on the left side of the spine, in a vertical position. The greater curvature looked towards the left side ; the lesser curvature towards the spine ; the pyloric orifice about one inch to the left of the spine, in a vertical line with the œsophageal extremity ; otherwise healthy ; small intestines healthy, but very small, and nearly empty. The ileum, instead of reaching the caput-coli in the right inguinal region proper, is seen running upwards as high as the middle of the right kidney, and terminating in the caput-coli, which is found in this region ; the vermiform process is found coiling around the iliac extremity of the right kidney. Large intestine healthy, but small ; mesentric glands healthy ; liver not enlarged ; gall-bladder and ducts, spleen and pancreas, and bladder, healthy ; kidneys slightly granular. On examining, and tracing the thoracic duct, from the abdomen, through the posterior mediastinum, to about the 2d dorsal vertebra, the coats were found very much thickened and opaque, and the calibre very much contracted ; it would easily admit a small iron knitting-needle for 3 to 4 inches ; it then became so narrow that the finest gold probe used for passing into the puncta lacrymalia, could with care be passed through it ; it soon became again dilated, so as to admit a knitting-needle as far as it could be passed without tracing it into the cervical region.

*Chest.*—Heart natural ; right lung do ; lower lobe of left lung in the 3d stage of Pneumonia (purulent infiltration, or yellow softening ;) upper lobe slightly congested ; no trace of tubercles. It was stated that the Doctor was 43 years old, and had been gradually emaciating for the last 18 years ; previous to which period he had weighed 133 lbs.; and that he now weighed only 45 lbs., and dated the commencement of his illness from having lain down on the damp ground. A brother and sister who are living, each of them weigh upwards of 200 lbs.

